# Telemedicine Practice, Perceived Barriers, and Concerns Among Doctors in Kerala

**DOI:** 10.7759/cureus.110697

**Published:** 2026-06-11

**Authors:** Anil Bindu Sukumaran, Manju Leelavathy

**Affiliations:** 1 Community Medicine, Sree Gokulam Medical College and Research Foundation, Thiruvananthapuram, IND

**Keywords:** barriers, concerns, digital health, ehealth literacy, health policy, teleconsultation, telehealth, telemedicine adoption, telemedicine practice

## Abstract

Telemedicine bridges gaps in accessibility and improves patient care. Its adoption has been progressively rising in India; however, there are considerable barriers to overcome. This study aimed to assess the utilization of telemedicine by doctors in Kerala and identify the perceived barriers and concerns faced by practicing doctors.

This cross-sectional study involved 535 doctors currently practicing modern medicine in Kerala. Practitioners of AYUSH systems (Ayurveda, Yoga and Naturopathy, Unani, Siddha, and Homeopathy) were excluded. A semi-structured questionnaire was used to collect data from June 2020 to May 2021. Participants were recruited using a nonrandom referral chain sampling method. Data analysis was carried out using IBM SPSS version 20.0 (IBM Corp., Armonk, NY). Chi-square tests were used as tests of significance. Among the participants, 337 (62.9%) doctors used telemedicine in their practice. Male gender (AOR=1.533; 95% CI: 1.028-2.288), availability of facilities for telemedicine practice in the hospital (AOR=3.032; 95% CI: 1.945-4.727), and attendance at a webinar or seminar (AOR=2.327; 95% CI: 1.52-3.542) were found to be associated with telemedicine use among doctors. The main barriers perceived by the doctors included the lack of direct physical contact with patients, 49 (9.2%); underuse of their clinical skills during telemedicine, 36 (6.7%); and lack of clarity regarding telemedicine regulations, 28 (5.2%).

Nearly two-thirds of the surveyed physicians reported using telemedicine in their clinical practice. Telemedicine practice was independently associated with male gender, availability of telemedicine facilities within the hospital, and participation in telemedicine-related educational activities. Despite its widespread adoption, physicians reported several barriers, including the inability to perform direct physical examinations, perceived underutilization of clinical skills, and lack of clarity regarding telemedicine regulations. Strengthening institutional telemedicine infrastructure, enhancing training opportunities, and improving awareness of regulatory guidelines may help address these challenges and support the sustainable integration of telemedicine into routine healthcare delivery.

## Introduction

Telemedicine, recognized as a vital technique with the potential to revolutionize healthcare globally, is particularly important in regions such as India. Because a large proportion of people in India reside in rural areas, telemedicine is important for overcoming disparities in healthcare access. The number of healthcare providers working in India is less than the World Health Organization (WHO)-recommended standard. Inequitable access to healthcare providers also exists [1-4]. Almost 60% of healthcare professionals cater to 27.8% of the urban population, while the remaining 72.2% of the rural population has access to only about 40% of the healthcare personnel [5]. A major milestone in the expansion of telemedicine in India was the publication of the Telemedicine Practice Guidelines in 2020, which provided a formal framework for telemedicine consultations by registered medical practitioners [6]. Registered medical practitioners were authorized to provide telemedicine services under these guidelines, but it was recommended that practitioners undergo telemedicine training through approved educational programs to familiarize themselves with the principles and requirements of telemedicine practice. After the introduction of the Telemedicine Practice Guidelines, government initiatives such as the eSanjeevani platform supported the large-scale implementation of telemedicine services throughout India. To maximize the advantages of telemedicine, barriers to its implementation must be addressed [7-9]. The eSanjeevani platform, introduced in 2020, has enabled over 466 million consultations. The telemedicine practice guidelines published in India have provided greater clarity among doctors and promoted this growth [10-12]. Digital health platforms were adopted by private healthcare organizations and clinicians to provide care through telemedicine during the lockdowns [13]. The adoption of telemedicine was not uniform across the country. Multiple barriers have been reported, including privacy and data security concerns, reluctance among clinicians who are less tech-savvy, inadequate infrastructure, and insufficient digital literacy skills. Telemedicine gained acceptance during the pandemic and has emerged as a major success in India; however, its sustained use is restricted by many issues [8,14-16]. Legal concerns, infrastructure deficiencies, network issues, restrictions on reimbursement, ease of use, and accessibility of patient records must be considered when implementing telemedicine. Inadequate resources are another problem that must be addressed in this regard. Better sustainability of telemedicine practice requires appropriate models [17]. Patterns in the adoption of telemedicine may differ depending on many factors. Gender dynamics may affect telemedicine practice [17,18]. The association between professional demographics, institutional characteristics, and physicians’ adoption of telemedicine has not yet been explored in Kerala.

Kerala, with its well-developed healthcare system, high literacy levels, favorable health indicators, and relatively high levels of digital connectivity, has been at the forefront of several digital health initiatives. Despite the growing adoption of telemedicine, limited evidence is available regarding telemedicine practices, barriers, and concerns among physicians in the state of Kerala. Hence, the objective of this study was to assess telemedicine utilization among doctors practicing modern scientific medicine in Kerala and to identify perceived barriers and professional concerns related to telemedicine implementation.

## Materials and methods

Study design and setting

This was a cross-sectional survey conducted in Kerala, India.

Study population

The study population included doctors practicing modern medicine.

Inclusion criteria

Doctors currently practicing modern medicine, defined as the contemporary scientific, evidence-based system of healthcare, were included.

Exclusion criteria

Practitioners of AYUSH systems (Ayurveda, Yoga and Naturopathy, Unani, Siddha, and Homeopathy) were excluded from the study.

Sample size and sampling technique

Based on a pilot study among 30 doctors, the sample size was calculated to be 171 (calculated using p=36%, with 20% relative precision). In order to improve precision and account for potential variability in responses, recruitment continued beyond the minimum requirement. We received 535 responses from doctors in Kerala between June 2020 and May 2021.

Non-random referral chain sampling was used. The Google Form link to the questionnaire was sent with an invitation to participate in the study through WhatsApp groups of doctors, Messenger (Facebook), or email. Doctors who received the questionnaire were requested to share it with their colleagues to recruit additional doctors through chain referral.

Data collection and study variables

This study represents a secondary analysis of data collected through a cross-sectional survey of physicians in Kerala. Findings related to telemedicine awareness and skills from this dataset have been previously reported [19]. The current analysis focuses on different research questions, specifically telemedicine practice, associated factors, perceived barriers, and professional concerns of physicians.

In this study, telemedicine was defined as the provision of clinical consultation, follow-up care, prescription advice, or patient communication through remote digital communication technology. This included telephone consultations, video consultations, text messaging services (e.g., SMS or messaging applications), email-based consultations, and consultations conducted through institutional telemedicine platforms. The doctors were asked to report the communication methods they commonly used in telemedicine practice.

A semi-structured questionnaire was developed by the investigators after reviewing the literature. It was reviewed by six experts. Modifications were made to the questionnaire based on their feedback regarding relevance, clarity, and comprehensiveness before data collection began. The survey included demographic and professional details and information on current telemedicine usage patterns through questions covering telemedicine practice and preferred communication methods. The doctors were asked whether dedicated telemedicine facilities were available in their hospitals and whether they had attended any telemedicine-related educational programs, such as webinars or seminars. These variables were recorded based on the participants’ self-reports. Questions on perceived barriers and concerns related to telemedicine implementation included closed-ended items for systematic barriers and open-ended questions for additional insights (Appendix 1). The online survey was configured to minimize duplicate responses. An informed consent statement was provided at the beginning of the online survey. Only participants who consented were asked to complete the questionnaire. The study was approved by the Institutional Ethics Committee (approval number: SGMCIEC No. 37/523/10/2020). The study variables included age, gender, qualifications, years of clinical experience, telemedicine use, preferred communication methods, availability of telemedicine facilities in hospitals, type of hospital, participation in educational programs related to telemedicine, perceived barriers, and concerns regarding telemedicine implementation. The completed questionnaires were reviewed for completeness prior to analysis. Duplicate entries were identified and removed by reviewing response timestamps and participant characteristics. Complete and unique responses were included in the final analysis.

Statistical analysis

Data were analyzed using IBM SPSS Statistics for Windows, version 20.0 (IBM Corp., Armonk, NY). Descriptive statistics (mean, standard deviation, frequency, percentage, median, and interquartile range) were used to summarize the data. The association between physician characteristics and telemedicine use was assessed using the chi-squared test. Multiple logistic regression was performed with telemedicine use as the dependent variable to identify factors independently associated with telemedicine utilization. Adjusted odds ratios (ORs) with 95% confidence intervals (CIs) were calculated. A p-value of <0.05 was considered statistically significant. Responses to the open-ended questions were independently reviewed by two investigators. They were grouped into themes based on content similarity. Closely related responses were combined into broader themes through discussion and consensus among the investigators.

## Results

The participants had a mean age of 40.44±10.7 years and a median of 12 years of clinical experience, with an interquartile range of 17. Most participants were from tertiary hospitals (406, 75.9%), followed by primary (70, 13.1%) and secondary hospitals (59, 11.0%). There were 462 (86.4%) doctors working in the private sector and 73 (13.6%) doctors employed in government healthcare institutions. A total of 389 (72.7%) doctors worked in teaching hospitals, and 146 (27.3%) worked in non-teaching hospitals. Among the doctors, 368 (68.8%) practiced in urban areas and 167 (31.2%) worked in rural areas. Doctors from all 14 districts of Kerala responded, with the largest proportion from Thiruvananthapuram (216, 40.4%), followed by Kollam (91, 17.0%), Ernakulam (49, 9.2%), and Alappuzha (38, 7.1%). Among the 535 doctors surveyed, 183 (34.2%) indicated that telemedicine services were available in their hospitals. Most physicians (325, 60.7%) had not attended any telemedicine-related seminar, webinar, or training in the preceding six months, while 74 (13.8%), 65 (12.1%), 19 (3.6%), and 52 (9.7%) had attended one, two, three, and more than three such educational activities, respectively. Among the doctors who participated, 337 (63.0%) reported that they used telemedicine for patient consultation.

Table 1 illustrates the relationship between physician characteristics and telemedicine practice. Attendance at educational programs such as webinars or seminars (p<0.001), availability of telemedicine facilities in the hospital (p<0.001), gender (p=0.008), and area of practice (urban/rural) (p=0.019) were significantly associated with the use of telemedicine in univariate analysis.

**Table 1:** Association between physician characteristics and telemedicine practice *p<0.05; **p<0.01; ***p< 0.001

Variables	Never practiced, N (%)	Practiced, N (%)	p-value
Age			
<30	42 (32.6)	87 (67.4)	0.753
30-40	61 (38.4)	98 (61.6)	
40-50	52 (36.9)	89 (63.1)	
50-60	33 (39.8)	50 (60.2)	
>60	10 (43.5)	13 (56.5)	
Gender			
Male	63 (30.1)	146 (69.9)	0.008**
Female	135 (41.4)	191 (58.6)	
Qualification			
Graduate	74 (37)	126 (63)	0.801
Postgraduate	114 (36.5)	198 (63.5)	
Super specialty	10 (43.5)	13 (56.5)	
Years of experience			
<5	63 (35.2)	116 (64.8)	0.681
05-10	22 (39.3)	34 (60.7)	
10-20	50 (36.2)	88 (63.8)	
20-30	42 (35.6)	76 (64.4)	
30-40	18 (50)	18 (50)	
>40	3 (37.5)	5 (62.5)	
Type of hospital:			
Primary level hospital	25 (35.7)	45 (64.3)	
Secondary level hospital	19 (32.2)	40 (67.8)	0.676
Tertiary level hospital	154 (37.9)	252 (62.1)	
Currently working in govt/private:			
Government	18 (24.7)	55 (75.3)	0.019*
Private	180 (39.0)	282 (61.0)	
Are you working in a teaching hospital?			
No	47 (32.2)	99 (67.8)	
Yes	151 (38.8)	238 (61.2)	0.157
Is there any dedicated telemedicine facility in your hospital?			
No	160 (45.5)	192 (54.5)	<0.001***
Yes	38 (20.8)	145 (79.2)	
Number of seminars/webinars/lectures on telemedicine have you attended during the last 6 months?			
	None	149 (45.8)	176 (54.2)	
≥1	49 (23.3)	161 (76.7)	<0.001***
Area of practice			
Rural	59 (35.3)	108 (64.70)	0.588
Urban	139 (37.80)	229 (62.20)	

Among the 535 physicians who responded, 304 (56.8%) reported sometimes using telemedicine for patient consultations, 33 (6.2%) reported using it often, while 198 (37.0%) reported never using telemedicine. Among the doctors in the study who reported practicing telemedicine (N=337), text messaging was the most preferred method for sending prescriptions to patients. Approximately 147 (43.6%) physicians used it for drug prescriptions. Another 124 (36.8%) preferred to share prescriptions as PDFs. Email was used by an even smaller proportion, 46 (13.6%) respondents, whereas only 20 (5.9%) reported using fax for prescription transmission. From the survey results, 325 (60.7%) doctors reported not having attended any seminar, webinar, or lecture on telemedicine in the six months preceding the survey.

A multivariable logistic regression model with telemedicine use as the dependent variable was performed. Variables that were found to be clinically significant and theoretically relevant based on the literature were included in the analysis. Variables such as age, gender, qualification, years of experience, type of current hospital, work environment (teaching/non-teaching), availability of facilities for telemedicine practice, and attendance at telemedicine education programs were used as independent variables. Having a dedicated telemedicine facility in the hospital increased the odds of using telemedicine among physicians (adjusted odds ratio (AOR)=3.032; 95% CI: 1.945-4.727). Attending telemedicine-related educational programs increased the likelihood of telemedicine use (AOR=2.327; 95% CI: 1.52-3.542). The odds of using telemedicine among males were significantly higher than those among females (AOR=1.533; 95% CI: 1.028-2.288). Other demographic and institutional factors were not significantly associated with telemedicine use (Table 2).

**Table 2:** Multivariable logistic regression analysis of factors associated with telemedicine use among physicians ***p<0.05; **p<0.01; *p< 0.001 OR, odds ratio

	p-value	OR	95% CI for OR	
Variable			Lower	Upper
Age				
<30 (Ref)				
30-40	0.127	0.597	0.307	1.159
40-50	0.227	0.547	0.206	1.454
50-60	0.499	0.652	0.189	2.251
>60	0.692	1.490	0.207	10.704
Gender				
Male				
Female (Ref)	0.035*	1.537	1.030	2.292
Qualification				
Graduate (Ref)				
Postgraduate	0.634	0.903	0.591	1.377
Super specialty	0.208	0.498	0.168	1.476
Years of experience (Ref)				
<5				
05-10	0.817	1.094	0.511	2.341
10-20	0.378	1.425	0.649	3.127
20-30	0.524	1.405	0.493	4.004
30-40	0.595	0.667	0.150	2.963
>40	0.515	0.437	0.036	5.281
Type of hospital				
Primary level hospital (Ref)				
Secondary level hospital	0.811	1.102	0.497	2.444
Tertiary level hospital	0.674	1.148	0.603	2.188
Currently working in				
Government (Ref)				
Private	0.074	0.566	0.303	1.057
Are you working in a teaching hospital				
No (Ref)				
Yes	0.282	0.749	0.443	1.268
Is there any dedicated telemedicine facility in your hospital				
No (Ref)				
Yes	<0.001***	3.024	1.938	4.718
Number of seminars/webinars/lectures on telemedicine have you attended during the last 6 months?				
0 (Ref)				
≥1	<0.001***	2.338	1.532	3.569
Area of practice				
Rural				
Urban	0.851	1.042	0.680	1.595

Several barriers to using telemedicine were perceived by the doctors who participated in the survey. Among the doctors, 49 (9.2%) mentioned that not being able to physically examine their patients was a barrier. Table 3 shows the barriers identified by the physicians.

**Table 3:** Perceived barriers to implementing telemedicine Participants were allowed to select multiple disadvantages; therefore, individual respondents may have been represented in more than one category.

Barriers	Frequency (%)
Financial	11 (2.1)
Privacy and security	19 (3.6)
Regulatory	28 (5.2)
Under usage of clinical skills	36 (6.7)
More time consumed than usual consultation	4 (0.7)
Patients' ease with the use of technology	6 (1.1)
Internet connectivity issues	13 (2.4)
Lack of awareness	32 (5.9)
Barriers reported through open-ended questions	
Doctor-patient connection poor	36 (6.7)
Poor technology knowledge	25 (4.7)
Legal implication	19 (3.5)
Not feasible	22 (4.1)
Accessibility	13 (2.4)
Not touching the patient	49 (9.2)
New assessment cannot be done and can be used only for chronic conditions	17 (3.1)
Fear of wrong diagnosis	15 (2.8)
Lack of follow-up	5 (0.9)
Misuse	7 (1.3)
Decrease in doctor skills	7 (1.3)
Others	54 (10.1)
None	210 (39.2)

Participants were allowed to select multiple options; therefore, individual respondents may have been represented in more than one category. The figure displays the frequencies of the different combinations of responses reported by the participants. Percentages were calculated using the total number of respondents (n=535) as the denominator.

**Figure 1:**
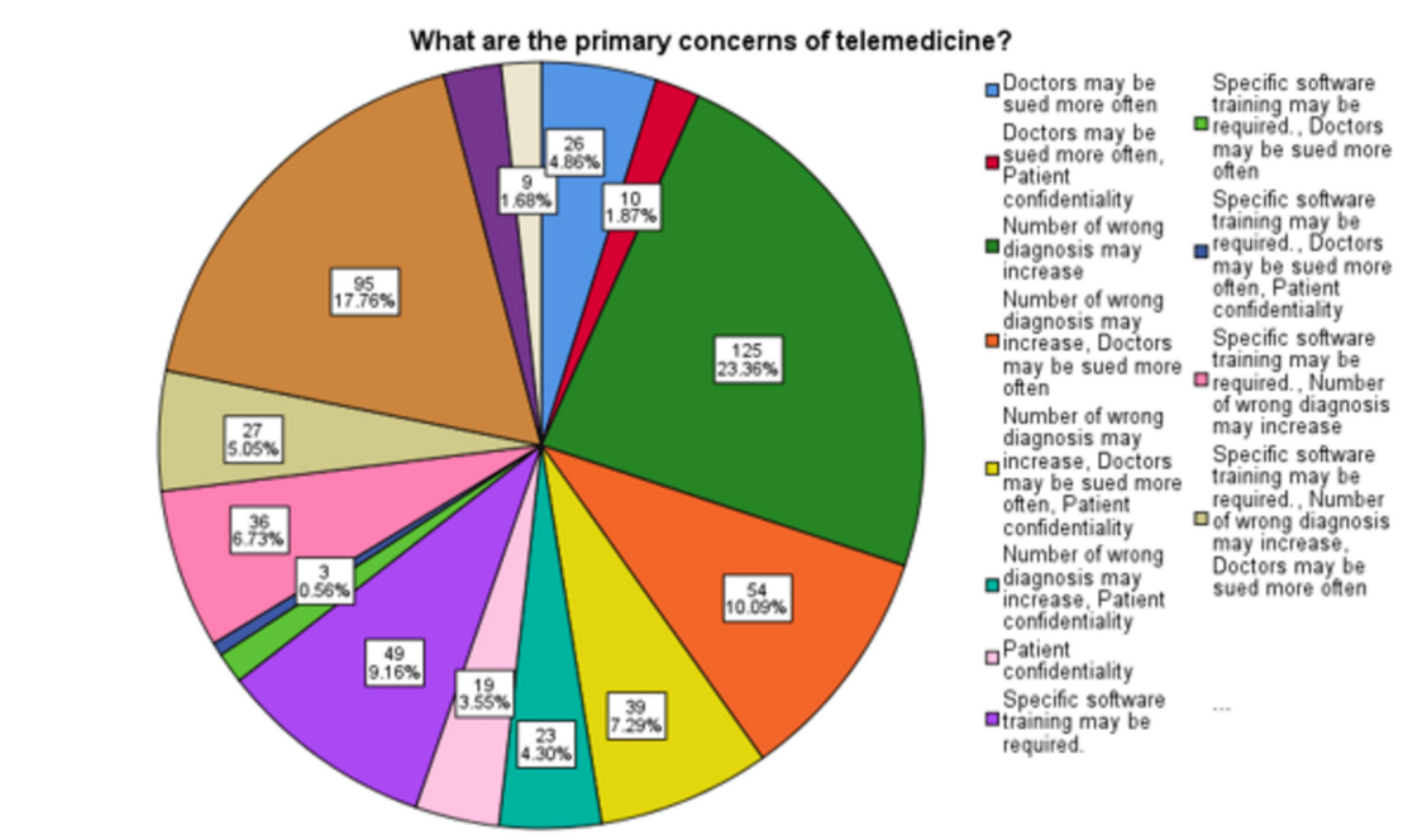
Primary concerns of doctors about telemedicine practice Participants were allowed to select multiple concerns; therefore, individual respondents may have been represented in more than one category. The figure displays the frequencies of the different combinations of responses reported by participants. Percentages were calculated using the total number of respondents (n=535) as the denominator.

Several concerns regarding the application of telemedicine were perceived by the doctors who participated in the survey. Figure 1 shows the distribution of various combinations of concerns expressed by doctors about telemedicine.

## Discussion

This cross-sectional study revealed that 337 (62.9%) modern medicine doctors had practiced telemedicine. This finding, showing 62.9% physician adoption, is consistent with the general trend of increasing teleconsultations observed via national platforms such as eSanjeevani [20]. Strong institutional support in alignment with telemedicine guidelines may further enhance telemedicine adoption among healthcare professionals [21]. Multivariable analysis showed that the availability of telemedicine facilities and participation in telemedicine-related educational programs were independently associated with telemedicine use among physicians, highlighting the importance of institutional support and training initiatives in promoting telemedicine utilization. Future efforts should focus on strengthening telemedicine infrastructure and integrating telemedicine-related training into continuing medical education programs.

Only 183 (34.2%) doctors in current practice reported that their hospitals had dedicated telemedicine facilities. However, 337 (62.9%) reported using telemedicine in clinical practice. This difference suggests that almost half of the doctors use personal devices and low-resource solutions instead of fully institutionalized platforms for telemedicine consultations. However, the present study did not specifically assess physician utilization of any particular platform. Another study conducted in India reported that live audio or video consultations (60.4%) were used for teleconsultations [22]. These findings suggest a strong need to institutionalize and standardize telehealth infrastructure to address the identified gaps. This study found that many doctors still use semi-informal digital settings outside the institutional framework, such as text messaging and sharing PDFs. This may create concerns regarding privacy, legal responsibility, and quality assurance, which were also reported by the participants. Improving hospital-supported telemedicine platforms can enhance telemedicine use by doctors, assist with documentation, and ensure compliance with regulations. This will make teleconsultations safer and more effective, and will also support the long-term integration of digital health.

In this study, the doctors reported many perceived barriers to adopting telemedicine practices. They faced hurdles comparable to those reported in current telehealth research studies [23-25]. The inability to perform physical examinations or engage with patients physically, reported by 49 (9.2%) physicians, was an important perceived barrier in this study. The findings of this study are in line with those reported at the national level, which also show that clinicians are concerned about not being able to perform physical examinations, diagnostic uncertainty, and missing important clinical signs. National surveys have similarly reported that physicians are concerned about conducting physical examinations remotely, are uncertain about diagnoses, and may miss crucial information. Inability to use clinical skills appropriately and poor doctor-patient interaction have also been recognized in other studies as major factors preventing doctors from using telemedicine services, especially for complicated or first-time consultations [10]. To address these challenges and enhance diagnostic accuracy while practicing telemedicine, the use of advanced digital health tools, such as high-resolution video platforms and digital stethoscopes, may prove advantageous.

Several technical and skill-based challenges faced by doctors were reported in this study, including limited technological knowledge and poor Internet connectivity. These findings are similar to those reported in previous studies conducted in India [23]. In Kerala, although doctors generally have a positive attitude toward telemedicine, many report inadequate familiarity with certain tools and applications, and significant human resource and workflow challenges [26]. It was found that 325 (60.7%) physicians had not participated in any educational programs dedicated to telemedicine. This suggests a lack of necessary training despite the country's telemedicine guidelines being in place. Targeted educational programs and training are required to address these gaps. To maximize the benefits of telemedicine, the healthcare system must ensure that doctors are confident and adequately trained in its use. Physicians are key stakeholders, and their concerns must be addressed. Many doctors who participated in this study expressed concerns regarding regulatory and ethical issues. These included concerns about the risk of litigation, fear of misdiagnosis, and data security issues. They also expressed the need for specialized software training to facilitate their work. Indian reviews and policy analyses strongly support these concerns. The 2020 Telemedicine Practice Guidelines and other policy documents attempted to establish clear regulations, but some areas remain unclear, especially regarding data storage standards and cybersecurity [21,27].

It is worth mentioning that 147 (43.6%) physicians opted for SMS-based prescriptions, whereas 124 (36.8%) preferred PDF-based prescriptions. This finding is consistent with the results of another study conducted in India that reported the use of cell phones by physicians in telemedicine consultations involving audio/video conferencing along with text-based communication for e-prescriptions and consultations [22]. While smartphones have made telemedicine easier and more rapid, they also pose challenges related to standardization, documentation, and data protection, particularly when general messaging services are used instead of specialized healthcare solutions. Improving institutional telemedicine platforms with integrated e-prescription modules and secure data storage could help address many of the privacy and medico-legal concerns raised by the participants.

The results of this study echo findings from the Malaysian healthcare system, where perceived ease of use and perceived usefulness were identified as pivotal factors in telemedicine adoption, and support recommendations that healthcare organizations and policymakers prioritize improving telemedicine infrastructure and interfaces to enhance perceived ease of use [28]. Tailored strategies that address country-specific contexts and healthcare system readiness are essential.

The finding that telemedicine adoption was higher among those who had attended telemedicine-related educational programs suggests that incorporating telemedicine training into continuing medical education, with greater emphasis on telemedicine protocols and legal considerations, may improve adoption. The unequal gender distribution of doctors utilizing telemedicine warrants further investigation to better understand the factors influencing telemedicine adoption among physicians.

This study was cross-sectional in nature, with a good sample size to evaluate physicians’ perspectives regarding telemedicine adoption in Kerala; however, non-random sampling techniques and online data collection using a self-administered questionnaire may limit the representativeness and generalizability of the findings to all physicians practicing in the state. Self-reporting bias or social desirability bias may have influenced the results. Also, the total number of doctors who received the survey invitation could not be determined, and the response rate could not be calculated. In addition, there may have been an overrepresentation of physicians who were more technologically engaged or interested in telemedicine than others. The findings of this study reflect telemedicine practices more broadly rather than experiences with any single telemedicine system. Differences in telemedicine use across specialties could not be evaluated in this study. This study represents a secondary analysis of a previously collected dataset, but it addresses a distinct research question that evaluates physician-reported practices, barriers, and concerns.

## Conclusions

Nearly two-thirds of doctors in Kerala have adopted telemedicine, but several issues continue to restrict its implementation. Physicians reported concerns such as the inability to perform physical examinations, legal and confidentiality concerns, and technological limitations that need to be addressed if telemedicine use is to be sustained. Targeted efforts to strengthen technological infrastructure, institutional support, and physician training are essential for the effective and sustainable integration of telemedicine into routine healthcare delivery.

## Appendices

Appendix 1 

**Table 4:** Questionnaire *Multiple responses were allowed.

Item no.	Question/item	Response options
1	What is your age (in years)?	Open-ended
2	What is your gender?	Male
		Female
		Other
3	What is your highest qualification?	Graduate
		Postgraduate
		Super-specialty
4	Currently working in government or private hospital	Government
		Private
5	Years of clinical experience	Open-ended
6	Type of hospital where you currently work	Primary-level hospital
		Secondary-level hospital
		Tertiary-level hospital
7	Are you working in a teaching hospital?	Yes
		No
8	Have you practiced telemedicine for consulting patients?	Yes
		No
9	How often do you consult patients using telemedicine?	Never
		Sometimes
		Often
10	How do you send prescriptions after telemedicine consultation?	Scanned prescription (PDF)
		Text message
		Email
		Fax
		Not practiced
		Others (specify)
11	Is there a dedicated telemedicine facility in your hospital?	Yes
		No
12	Number of telemedicine-related seminars/webinars attended in the last 6 months	0
		1
		2
		3
		More than 3
13	What are the barriers in implementing telemedicine in clinical practice?*	Regulatory barriers
		Financial barriers
		Privacy and security concerns
		Reduced clinical skills
		Internet connectivity issues
		Patient difficulty using technology
		Time-consuming
		Lack of awareness
14	Any other barriers (please specify)	Open-ended
15	What are your concerns about practicing telemedicine?*	Increased risk of wrong diagnosis
		Increased medico-legal risk
		Patient confidentiality concerns
		Requires specific software training
16	District of practice	Open-ended
17	Area of practice	Rural
		Urban
